# Modeling the impact of COVID‐19 nonpharmaceutical interventions on respiratory syncytial virus transmission in South Africa

**DOI:** 10.1111/irv.13229

**Published:** 2023-12-10

**Authors:** Samantha J. Bents, Cécile Viboud, Bryan T. Grenfell, Alexandra B. Hogan, Stefano Tempia, Anne von Gottberg, Jocelyn Moyes, Sibongile Walaza, Chelsea Hansen, Cheryl Cohen, Rachel E. Baker

**Affiliations:** ^1^ Fogarty International Center, National Institutes of Health Bethesda Maryland USA; ^2^ Department of Ecology and Evolutionary Biology Princeton University Princeton New Jersey USA; ^3^ School of Population Health University of New South Wales Sydney New South Wales Australia; ^4^ Centre for Respiratory Diseases and Meningitis National Institute for Communicable Diseases of the National Health Laboratory Service Johannesburg South Africa; ^5^ School of Public Health, Faculty of Health Sciences University of Witwatersrand Johannesburg South Africa; ^6^ School of Pathology, Faculty of Health Sciences University of Witwatersrand Johannesburg South Africa; ^7^ Department of Pathology, Faculty of Health Sciences University of Cape Town Cape Town South Africa; ^8^ Brotman Baty Institute University of Washington Seattle Washington USA; ^9^ PandemiX Center, Department of Science & Environment Roskilde University Roskilde Denmark; ^10^ School of Public Health Brown University Providence Rhode Island USA

**Keywords:** COVID‐19, epidemiological models, low‐ and middle‐income countries, respiratory syncytial virus

## Abstract

**Background:**

The South African government employed various nonpharmaceutical interventions (NPIs) to reduce the spread of SARS‐CoV‐2. Surveillance data from South Africa indicates reduced circulation of respiratory syncytial virus (RSV) throughout the 2020–2021 seasons. Here, we use a mechanistic transmission model to project the rebound of RSV in the two subsequent seasons.

**Methods:**

We fit an age‐structured epidemiological model to hospitalization data from national RSV surveillance in South Africa, allowing for time‐varying reduction in RSV transmission during periods of COVID‐19 circulation. We apply the model to project the rebound of RSV in the 2022 and 2023 seasons.

**Results:**

We projected an early and intense outbreak of RSV in April 2022, with an age shift to older infants (6–23 months old) experiencing a larger portion of severe disease burden than typical. In March 2022, government alerts were issued to prepare the hospital system for this potentially intense outbreak. We then assess the 2022 predictions and project the 2023 season. Model predictions for 2023 indicate that RSV activity has not fully returned to normal, with a projected early and moderately intense wave. We estimate that NPIs reduced RSV transmission between 15% and 50% during periods of COVID‐19 circulation.

**Conclusions:**

A wide range of NPIs impacted the dynamics of the RSV outbreaks throughout 2020–2023 in regard to timing, magnitude, and age structure, with important implications in a low‐ and middle‐income countries (LMICs) setting where RSV interventions remain limited. More efforts should focus on adapting RSV models to LMIC data to project the impact of upcoming medical interventions for this disease.

## 1 INTRODUCTION

Respiratory syncytial virus (RSV) is the most common cause of lower respiratory infections and is responsible for an estimated 5% of under‐five mortality globally.^1^ The majority of RSV disease burden is concentrated in low‐resource settings, with 97% of all RSV‐related deaths occurring in low‐ and middle‐income countries (LMICs).^2^ Almost all children are infected by the age of 2 years, but severe infections occur primarily in infants under 6 months of age.^3^, ^4^, ^5^ Given the short waning time of RSV‐specific antibodies, reinfections occur frequently at older ages, but secondary infections are unlikely to result in hospitalization.^3^ Recent clinical trials have shown promising results for maternal RSV vaccines and long‐lasting monoclonal antibodies to avert medical illnesses in high‐risk infants, yet none of these interventions are currently available in LMIC.^6^, ^7^

While transmission is driven primarily by exposure between young children in high‐contact settings such as schools, there is strong seasonal forcing in RSV activity modulated in part by low specific humidity.^8^, ^9^ The COVID‐19 pandemic has disrupted the typical seasonality of RSV, with many countries reporting historically low transmission of RSV in periods of high nonpharmaceutical interventions (NPIs), followed by large, out‐of‐season outbreaks as NPIs were lifted.^10^, ^11^, ^12^, ^13^, ^14^ Here, we model RSV dynamics in the pre‐ and post‐pandemic period in South Africa, where the availability of robust RSV surveillance data provides a unique opportunity to understand disease dynamics in a lower middle‐income country setting. We focus on the hospitalization impact of the RSV rebound in the post‐pandemic period, as RSV mitigation measures are very limited and hospital surge capacity is heavily constrained in South Africa. We also study demographic shifts in the profile of affected children in the rebound period.

By mid‐2022, South Africa had experienced five major waves of SARS‐CoV‐2 infection driven by different variants, with varying levels of NPIs in place (Figure S1).^15^ These NPIs have affected population behavior and reduced circulation of a range of communicable respiratory diseases, with record‐low RSV and influenza seasons reported in 2020.^15^, ^16^ As NPIs were relaxed in the Southern Hemisphere spring of 2020, RSV made a late but historically low out‐of‐season resurgence that persisted into 2021.^15^ We discuss a first round of RSV projections delivered during the 2022 season (presented as a preprint in Bents et al.^17^), and an updated model fit and forecasts for the upcoming 2023 season. This work demonstrates the utility of epidemiological modeling in providing insight during periods of uncertainty, as exemplified by perturbations due to the COVID‐19 pandemic, and in providing a baseline to compare the impact of future medical interventions for RSV.

## 2 METHODS

### 2.1 Epidemiological data

This study uses facility‐based data collected through the Severe Acute Respiratory Illness (SARI) Surveillance Program from January 2016 to March 2023. SARI surveillance is conducted among inpatients in seven hospitals spanning five provinces: Western Cape, North West, Gauteng, KwaZulu‐Natal, and Mpumalanga. Respiratory specimens collected among enrolled children meeting the clinical case definition for a lower respiratory tract infection using nasopharyngeal aspirates or nasopharyngeal and oropharyngeal swabs. Positives tests are confirmed using real‐time reverse transcription PCR (RT‐PCR) and subsequently reported.^15^ The enrolled population ranges in age from <1 month to 91 years of age. We disaggregate the data into fine groups for young children (0–2 months, 3–5 months, 6–11 months, 12–23 months, and 2+ years) to focus on RSV burden in a highly impacted population.

### 2.2 Transmission model

We use an age‐structured Susceptible–Exposed–Infectious–Recovered–Susceptible (SEIRS) compartmental model originally described by Hogan et al. to model RSV in Western Australia^18^ and adapt it to South Africa. We fit the model to age‐structured hospitalizations reported through SARI surveillance since 2016, as we were interested primarily in the burden of severe disease. We aggregated weekly reported hospitalizations among all ages to a monthly scale to align with the model structure. The full SEIRS model is given by

$$\frac{dS_{i}}{\mathrm{dt}}=-\lambda_{i}\omega_{j}S_{i}+vR_{i},$$

$$\frac{dE_{i}}{\mathrm{dt}}=\lambda_{i}\omega_{j}S_{i}-\delta E_{i},$$

$$\frac{dI_{i}}{\mathrm{dt}}=\delta E_{i}-\gamma I_{i},$$

$$\frac{dR_{i}}{\mathrm{dt}}=\gamma I_{i}-vR_{i},$$

where *S* is the susceptible population, *E* is exposed, *I* is infectious, *R* is recovered, and *i* represents a given age cohort (monthly age cohorts for children ≤5 years and 5‐year age cohorts for persons aged 5–75 years). 1/*γ* is the infectious period, which is set to 9 days, and 1/*δ* is the latent period, which is set to 4 days (see Table 1). 1/*ν* is the immunity period which is estimated from the data. *ω*_*j*_ represents reduced infectiousness in children older than 10 years and is fixed from prior work.^18^

**TABLE 1:** Model parameters and parameter ranges.

Parameter	Definition	Range of estimates used in calibration step	Estimate or fixed value
*v*	Duration of immunity (days)	200–300	227 (95% CI: 217, 236)
*β*_1_	Amplitude of seasonal forcing	0–1	0.245 (95% CI: 0.211, 0.282)
*γ*	Infectious period (days)	Fixed^18^	9
*δ*	Latent period (days)	Fixed^18^	4
ϕ	Phase shift	0–2*π*	2.011 (95% CI: 1.866, 2.168)
*β*_0_	Transmission coefficient	0–1	0.033 (95% CI: 0.031, 0.035)
*h*_1_, *h*_2_, *h*_3_, *h*_4_, *h*_5_	Scaling factors representing the risk of (reported) hospitalization given infection for 0–2 months, 3–5 months, 6–11 months, 12–23 months, and 2+ years	0–0.5	0.073 (95% CI: 0.065, 0.082) 0.020 (95% CI: 0.017, 0.023) 0.010 (95% CI: 0.008, 0.012) 0.003 (95% CI: 0.0027, 0.0035) 0.00004 (95% CI: 0.00003, 0.00005)
*ω*	Reduced infectiousness in >10 years old	Fixed^18^	0.6
*σ*_1_, *σ*_2_, *σ*_3_	Reduced infectiousness in >3 months old	Fixed^18^	0.08, 0.45, 0.45

*Note*: Fixed model parameters and the range of estimates considered for each parameter fit to surveillance data.

The RSV force of infection on a susceptible in age class *i* is given by

$$\lambda_{i}\left(t\right)=\beta_{0}\left(1+\beta_{1}\cos\left(\;\frac{2\mathrm{\pi t}}{12}+\phi \right)\right)\frac{1}{N_{i}}\sum_{j=1}^{75}\;M_{i,\;j}\;\omega_{j}I_{j}.$$

Here, *t* is time in months, *λ*_*i*_(*t*) is the monthly transmission rate, and *β*_1_ and ϕ represent the amplitude and phase shift, respectively, that capture the seasonality in transmission in South Africa, to be estimated from the data. *M*_*i*,*j*_ describes the contact matrix between individuals in age groups *j* and *i*; we use an expanded version of the contact matrix originally described by Mossong et al., which describes population mixing patterns for Great Britain.^19^ We tried using a synthetic South African‐specific contact matrix, but it did not fit the data as well, possibly because the South African matrix is more appropriate for rural parts of the country while our RSV sentinel surveillance sites are based primarily in urban communities.^20^ Cohort aging is used to simulate monthly movements between age classes where individuals from each compartment are shifted instantaneously at fixed time points in the simulation.^21^, ^22^ This method of age class movement assumes a constant birth rate over time, aligning with the relatively stable birth rates observed in South Africa over our study period. We assume maternal immunity reduces disease susceptibility in the first 3 months of life.^23^, ^24^ In line with a seroepidemiological study from Brazil, maternal immunity reduces susceptibility to infection by 92% in the first month of life and by 55% in the next 2 months of life.^18^, ^25^

### 2.3 Calibration process

We use a two‐step process to fit the pre‐pandemic and pandemic periods. In the first step, we fit the core model described above to surveillance data from the pre‐pandemic period, running from January 2016 to December 2019. In the second step, we refit the model to a longer dataset that includes the pandemic period, keeping the same core parameters as in the first step but allowing for reductions in RSV transmission during the pandemic period, to be estimated from the data (see below).

In the first step, we simultaneously fit the seasonal coefficients *β*_0_, *β*_1_, ϕ, and immunity parameter *v* to pre‐pandemic observations using maximum likelihood parameter estimation (Figure S2). Projected model infection incidences are scaled to SARI surveillance data by fitting hospitalization scalars for infants 0–2 months, 3–5 months, 6–11 months, 12–23 months, and 2+ years. We calculate the average monthly time series of hospitalizations in each of these age groups from 2016 to 2019 surveillance data and use maximum likelihood estimation to calculate the optimal hospitalization rates that scale modeled infections to the average reported hospitalizations in the five relevant age groups. We estimated hospitalization scaling factors to be 0.073, 0.020, 0.010, 0.003, and 0.00004 for the five respective age groups (*h*_1_, *h*_2_, *h*_3_, *h*_4_, and *h*_5_ in Table 1).

We estimated the total population size modeled by summing the populations of the cities surrounding the hospitals included in the surveillance system as true catchment size was not available. We acknowledge that this is likely an overestimation of the actual catchment size, and estimations for true RSV incidence in South Africa are scarce. Therefore, the hospitalization scalars calculated should not be interpreted as the true risk of hospitalization given RSV infection but instead, as model scalars that are a product of both the age‐dependent severity process and the unknown surveillance catchment size and reporting rate of hospitalizations.

Finally, we use data from the pandemic period to estimate how control measures implemented to prevent the spread of COVID‐19 have affected RSV transmission. The pandemic period runs from January 2020 to December 2022 (spanning all COVID‐19 waves including Omicron and related lineages). We measure the time‐varying strength of the control periods with *c*(*t*), the percent reduction in transmission *λ*_*i*_(*t*). The adjusted equation for the force of infection then can be described as

$$\lambda_{i}\left(t\right)=c\left(t\right)\beta_{0}\left(1+\beta_{1}\cos\left(\;\frac{2\mathrm{\pi t}}{12}+\phi \right)\right)\frac{1}{N_{i}}\sum_{j=1}^{75}M_{i,\;j}\omega_{j}I_{j},$$

where 0 < *c*(*t*) < 1. Given the diverse array of NPI recommendations and behavioral compliance, we allow for a dynamic reduction in transmission by iteratively fitting *c*(*t*) in 2‐month intervals from January 2020 to December 2022, allowing for 18 distinct NPI periods. We optimize *c*(*t*) throughout the pandemic period by minimizing the mean squared error between modeled and observed hospitalizations and find that *c*(*t*) is greatest during periods of stringent NPI and sustained COVID‐19 circulation (Figure S1).

After calibrating the model to pre‐pandemic data and estimating the impact of 2020–2022 interventions, we let the model run during 2023–2024 to project RSV trajectory into future years. Our main results are based on these projections, particularly the projected timing, amplitude, and age distribution of RSV hospitalizations in the 2023 season. We also present a set of hindcasts for the 2022 season, based on fitting interventions for the January 2020–March 2022 period and letting the model run forward. These hindcasts are based on earlier projections that were used in real time to alert hospitals in South Africa ahead of the 2022 season, as documented in a preprint.^17^

## 3 RESULTS

### 3.1 RSV patterns in 2020–2022 surveillance data

**FIGURE 1:**
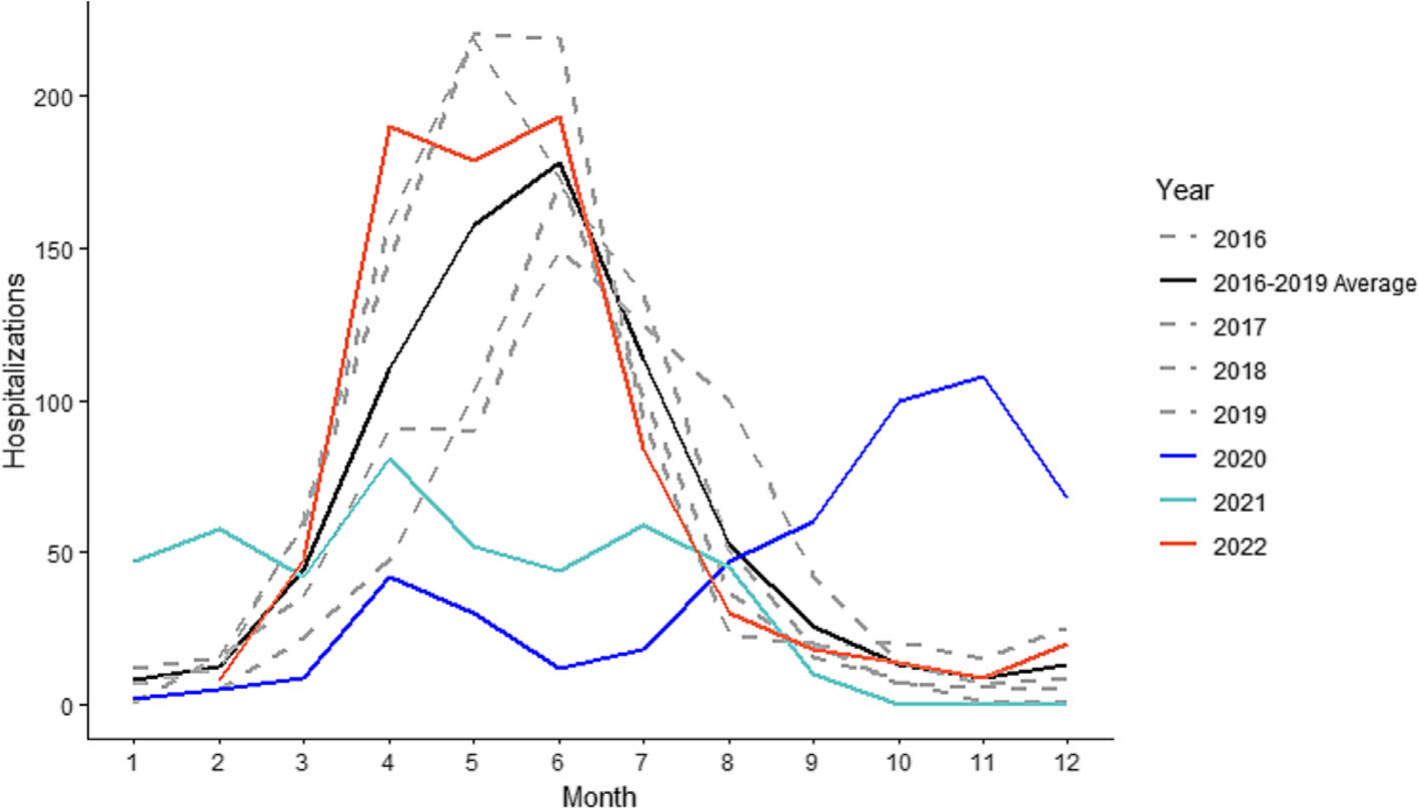
Reported RSV hospitalizations, South Africa 2016–2022. The number of monthly hospitalizations reported through SARI surveillance for the 2020 season, 2021 season, and 2022 season compared with the average for five preceding seasons, which follows the typical seasonal transmission pattern. Individual years 2015–2019 show typical seasonal variability.

The NPIs implemented to control COVID‐19 caused dramatic disruptions in RSV transmission patterns from 2020 to 2021 as shown by Figure 1. RSV was almost completely suppressed during the typical transmission period in 2020 but made a strong resurgence in August–December of 2020, coinciding with the relaxation of NPIs. Transmission continued into 2021 following normal seasonal patterns but was reduced as a result of the implementation of stricter NPIs to control the second COVID‐19 wave in South Africa (Beta variant). Overall, the 2020–2021 seasons showed historically low transmission, with the 2020 and 2021 outbreaks recording approximately 68.7% and 60.1% of the number of hospitalizations relative to the 2016–2019 pre‐pandemic average.

### 3.2 RSV model projections

**FIGURE 2:**
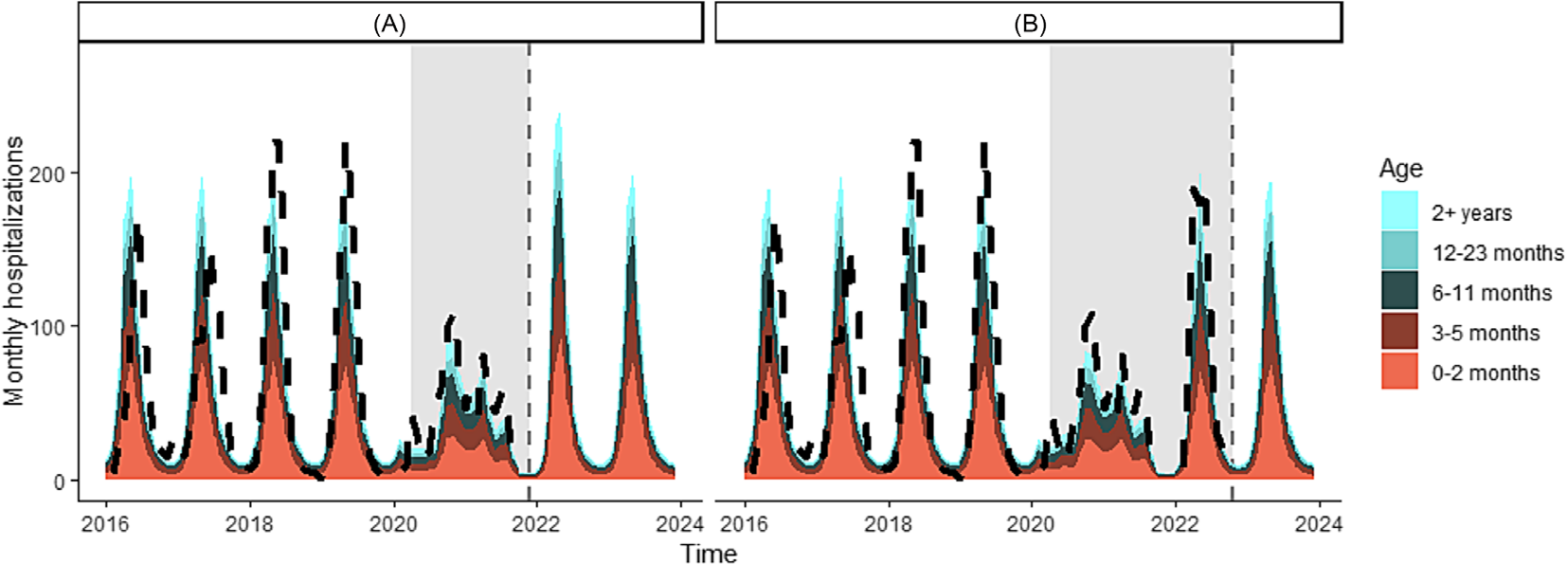
Age‐structured model projections and observations of the monthly number of national RSV hospitalizations in South Africa at two time points. Model projections are represented in color, with different age groups represented based on the color bar on the right. The black dashed line represents observed hospitalizations as reported through SARI and the gray vertical line shows the end of the calibration data. Gray bars show the NPI period. (A) Model fit and prediction for the 2022 RSV reason in which we assumed that no NPIs would be in place after October 2021. (B) Model updates based on calibration to the 2022 RSV season and prediction for the 2023 season.

In a prior version of this work published in early 2022,^17^ we had predicted a 32% increase in the number of monthly hospitalizations at the peak of infection in 2022 and a shift in the age structure to older individuals (>12 months) experiencing more severe illness (Figure 2A). This prior work did not consider any NPI during the Omicron period. We retrospectively assessed these predictions with updated surveillance data and found that an early and intense outbreak of RSV was observed in 2022 but at a lower intensity than predicted by our analysis. There was considerable variation between projections and observations by month due to slight differences in timing, but the projections delivered in early 2022 were on average 8.1% (95% CI: −28.6, 44.8) higher than those delivered after the model had been updated with 2022 surveillance data.

In our next set of projections, we incorporate updated surveillance data until the end of 2022 into the model to project the 2023 season (Figure 2B). The 2023 outbreak is estimated to be similar in size to a large, pre‐pandemic outbreak with a peak in April 2023. Compared with 2022 predictions, the model estimates a less intense peak size in 2023 (6.4% increase in intensity compared with pre‐pandemic seasons) but a slightly larger overall hospital burden.

Here, we display projections for the 2023 season in which we fit a 2‐month moving reduction in transmission from January 2020 to December 2022 alongside the initial projections delivered in March 2022 for the 2022 season (Figure 2). In the updated analysis considering the impact of interventions throughout 2020–2022, we find that reductions in RSV transmission range from 0% to 50% throughout the fitting period (Figure S1). The largest reductions in transmission occur in early 2020 and mid‐2021, coinciding with periods of stringent NPI and sustained COVID‐19 transmission. During the Omicron peak from December 2021 to February 2022, reduction in transmission does not exceed 15%, likely reflecting the lack of stringent NPIs in place by early 2022 despite high COVID‐19 circulation.

### 3.3 Demographic shift of hospitalizations

**FIGURE 3:**
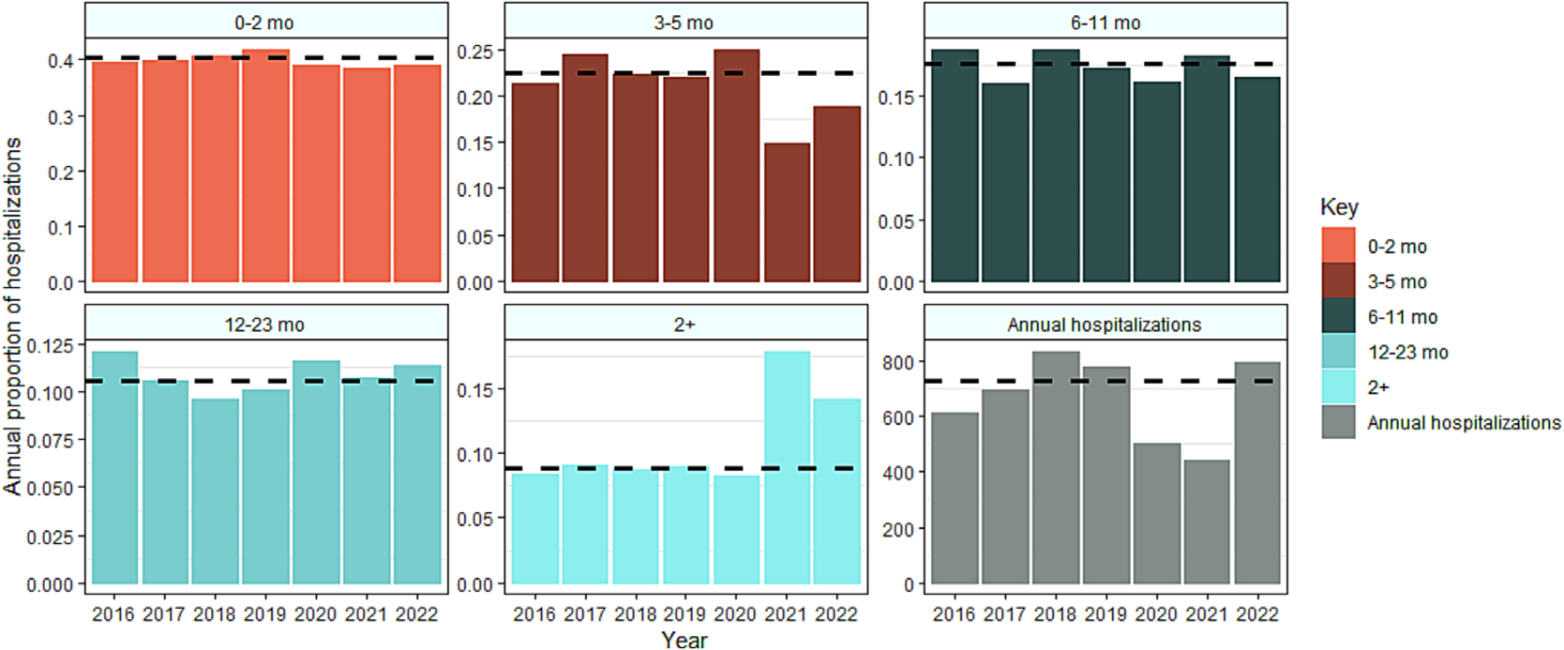
Age distribution and number of infants hospitalized with laboratory‐confirmed RSV January 2016–December 2022, sentinel surveillance, South Africa. The first five panels represent the age shift in RSV surveillance data coinciding with the COVID‐19 pandemic. Each panel represents the proportion of annual hospitalizations observed in the 0–2‐month, 3–5‐month, 6–11‐month, 12–23‐month, and 2+‐year age groups alongside annual burden reported from 2016 to 2022. The last panel represents the total number of hospitalizations across all age groups by year. The dashed black line indicates the pre‐pandemic average for each panel.

**FIGURE 4:**
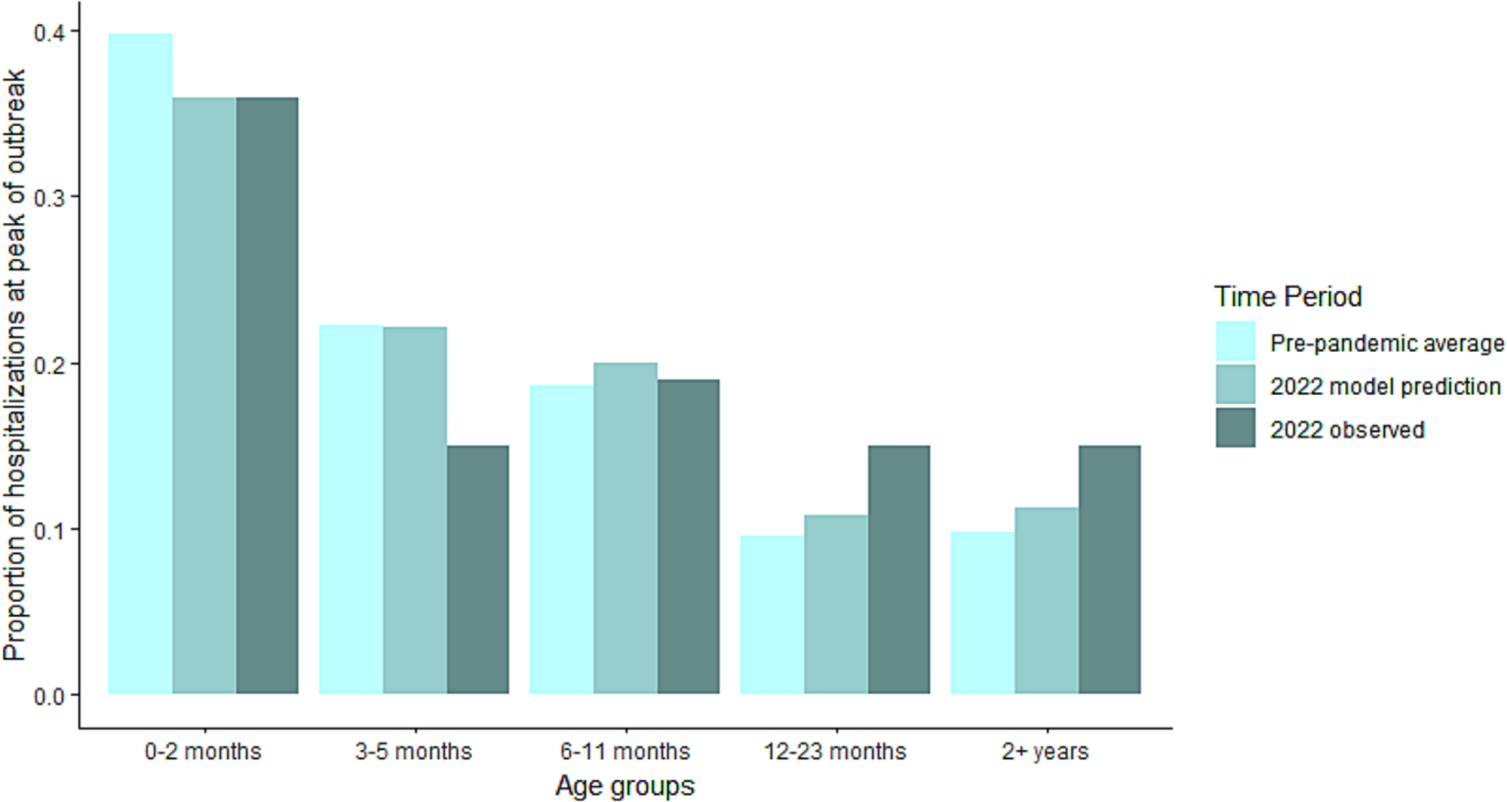
Modeled age structure. Predictions for the age structure at the peak month of the 2022 season compared with the modeled pre‐pandemic average and the 2022 observed season. The model generally captures the age shift observed in empirical data, with infants <6 months comprising a smaller proportion of the hospital burden. The model predicts the largest proportional increase in hospitalizations to be among children >12 months.

Age‐structured surveillance data show that the disruption in RSV transmission affected the age distribution of severe infections compared with pre‐pandemic years. Figure 3 shows the proportion of hospitalizations within the studied age groups (0–2 months, 3–5 months, 6–11 months, 12–23 months, and 2+ years) every year from 2016 to 2022. We observe a shift from 3–5 months to 2+ years that is most dramatic in 2021 and persists into 2022 (Figure 3). On average, infants 3–5 months old comprise 22.5% of annual hospitalizations, but this fell to 14.8% and 18.9% in 2021 and 2022, respectively. Conversely, the proportion of hospitalizations experienced by individuals >2 years nearly doubled over the same period. Further inspection of the data revealed this increase was most pronounced in the 3–4‐year‐old age group among children. The model was able to broadly capture the shifts in age structure observed in 2022, predicting the largest proportional increase in hospitalizations to be within individuals >12 months old (Figure 4).

## 4 DISCUSSION

We have calibrated an RSV transmission model to multiple years of hospitalization data in South Africa and shown the impact of NPIs implemented during the COVID‐19 pandemic on RSV transmission. Both the model and data indicate marked perturbations to RSV dynamics that had not fully resolved by early 2023. Our modeling showed that during 2020–2021, NPIs reduced RSV transmission by up to 50% during periods of high COVID‐19 circulation, while masking and behavioral changes in response to the Omicron surge limited transmission more moderately by 15% into 2022. These perturbations generated an early and intense outbreak with a shifted age distribution in 2022. We project that the 2023 season will have a lower peak than in 2022 but a slightly larger overall hospital burden, in part driven by the disappearance of pandemic NPIs.

Our initial modeling showed that the period of depressed RSV circulation in 2020–2021 could have contributed to creating the conditions favorable for producing a substantially intense 2022 RSV outbreak, posing the threat of overwhelming hospital capacity. Due to this possibility, the National Institute for Communicable Diseases of South Africa issued a government alert warning the public health system in early March 2022.^17^ Clinicians were informed of the possible increased numbers of RSV‐related hospital admissions and the importance of having sufficient available oxygen supplies and surge capacity plans. Hospitals were also warned to consider preparing for a larger number of babies experiencing severe primary infections at older ages than typical following two abnormally low RSV seasons. These pessimistic projections did not fully come to pass as we now know that low levels of NPIs remained in place during the Omicron wave, mitigating in part the 2022 RSV season. Even despite these low levels of NPIs, South Africa still observed an early and atypically large peak in the 2022 RSV season. This demonstrates the utility of epidemiological modeling in preparing healthcare systems for potentially high disease burden in times of uncertainty.

This modeling study also shines light on the importance of capturing the heterogeneous impacts of NPIs and associated behavioral compliance. In previous iterations of this work,^17^ the model implementation of NPIs was in line with national government alerts and failed to acknowledge the influence of behavioral compliance to these measures. For example, although South Africa remained at the lowest alert level^1^ from October 2021, an optional mask mandate remained in place in academic settings until June 2022, spanning two Omicron waves and the peak of the RSV season. Because schools are thought to be heavily implicated in the spread of childhood infections such as RSV, consistent masking in these high‐contact settings likely moderately lowered the intensity of the predicted peak, despite the government being at the lowest alert level. Additionally, behavioral changes in response to high rates of Omicron variant infection may have lowered typical social interaction levels, further reducing RSV circulation. In the updated analysis, we fit a dynamic reduction in transmission and find that it more accurately predicts the 2022 RSV season than solely imputing NPI periods in line with government alerts. We find that the same government measures can translate to different reductions in RSV transmission at different time points, suggesting that capturing behavioral compliance is essential in order to more accurately estimate the impact of NPIs.

Surveillance data from the pandemic period also provides novel insight into predictors for disease severity. The trend towards older infants being hospitalized in 2021–2022 suggests that the probability of severe RSV infection is influenced not only by age but also by prior immunity. Previous research has deliberated whether older infants (>6 months) are better protected from developing severe RSV infections due to lung maturation associated with aging or protective immunity from prior infection.^3^, ^26^, ^27^ Infants >6 months olds are typically less likely to experience severe infection, but reduced RSV circulation in the two preceding RSV seasons likely increased the number of older infants susceptible to primary infection.^25^ This finding suggests that while RSV severity is likely a feature of both age and prior exposure to the virus, immunologically naive children can experience elevated risk for severe infection even as they age >6 months. Although our model cannot fully disentangle the contribution of age versus number of prior infections in driving disease severity, other studies have supported this finding and reported older infants experiencing higher than average rates of RSV‐related hospitalization following the lifting of COVID‐19 NPIs.^28^, ^29^

More research on this topic is warranted, especially as this question has bearing on the impact of new RSV interventions that will alter the age‐specific risk of severe disease.^30^ Further, it is uncertain at this point whether the impact of the COVID‐19 pandemic has fully subsided. Residual gaps in population immunity to RSV and/or low levels of NPIs such as masking could continue to alter transmission compared with pre‐pandemic years. This could affect the perceived benefits of new RSV control measures, including long acting monoclonals and vaccines. Our modeling approach could provide a tool to help disentangle the long‐term impacts of NPIs and new RSV interventions, particularly in LMIC settings where the date of introduction and coverage levels of these interventions will vary greatly.

More broadly, countries around the world such as France, Japan, and Australia, among others, had previously reported large, out‐of‐season RSV outbreaks following the lift of COVID‐19 NPIs, reflecting a global pattern in the nature of RSV resurgence in response to pandemic‐related public health measures.^10^, ^14^, ^31^, ^32^ The potential for COVID‐19 NPIs to influence large outbreaks is important to consider in the context of a LMIC, in which infants tend to be at higher risk for developing severe symptoms upon RSV infection and diagnostic tests may be limited.^33^, ^34^ Given the high cost of current prophylaxis treatments such as Palivizumab, the potential hospital burden of an intense RSV outbreak may have been considerable in South Africa.^35^ While newer and less costly RSV interventions are on the horizon, none of these were available to any country during the COVID‐19 pandemic period to mitigate the rebound of RSV.

Hospitals have also been dually impacted by the downstream effects of reduced health‐seeking throughout the pandemic. From March 2020 to September 2021, total hospital admissions dropped by 60% in South Africa, indicating that non‐COVID‐19 illnesses have been both reduced and gone increasingly untreated in recent years.^16^ This suggests that there may be a surge in individuals needing hospital care as general health‐seeking returns to pre‐pandemic levels. While NPIs have been effective in limiting COVID‐19 transmission on a global scale, further studies should continue to explore the indirect effects of these public health measures on the transmission dynamics of other infectious diseases and related severe outcomes. This is especially important to consider in the context of an LMIC and suggests that access to pharmaceutical COVID‐19 prevention and treatments such as vaccines and prophylaxis should be prioritized as mitigation measures.

There are several caveats to our model predictions. First, non‐COVID‐19 disease surveillance systems generally underreported throughout the early months of the pandemic, suggesting that the low RSV transmission periods observed through SARI surveillance were potentially a product of reduced reporting in addition to decreased social mixing patterns, although the systematic program structure with dedicated staff screening all admissions may have mitigated this.^36^ Our modeling analysis focuses on severe infection among young children, which is more likely to continue to be reported during healthcare disruptions than mild infections, suggesting this effect is likely minimal. Second, we use an age‐contact structure derived from European data in our model; although some local data exist from South Africa, the fit of the model using this synthetic contact matrix was not as good (not shown). Further, the model used does not distinguish between primary and secondary infections in relation to transmissibility and severity, but by fitting the age‐structured model to age‐structured hospitalizations, we show that the model captures the distribution of severe infections among the studied age groups. Lastly, the model does not consider RSV‐A and RSV‐B subtype‐specific dynamics, as serotype‐specific virological data are scarce. While the atypical timing of the RSV rebounds in South Africa and elsewhere suggests that NPIs considerably influenced the patterns observed, it is possible that subtype cycling additionally influenced the transmission dynamics or age structure of the 2022–2023 outbreaks.

## 5 CONCLUSIONS

We have shown how the COVID‐19 pandemic has perturbed the long‐term dynamics of RSV in South Africa, as in many other countries, due to the long‐lasting effect of NPIs. While the 2022 season could have been quite burdensome on the hospital system, masking mandates and reactive behavioral changes in response to the Omicron surge may have helped modulate transmission and reduce hospitalization loads to near typical pre‐pandemic levels. After retrospectively fitting the model to 2022 hospitalization data, we forecast the 2023 season and find that the model predicts another strong outbreak, suggesting that NPIs influenced three consecutive years of RSV activity. We observed a shift towards older infants being hospitalized at higher rates throughout the pandemic period, driven by an increase in susceptibility at older ages from reduced RSV circulation during the COVID‐19 pandemic. Overall, further work is warranted to understand the long‐lasting impacts of the COVID‐19 pandemic on the transmission and severity of a range of pathogens globally, including those with a strong concentration in very young age groups that are less affected by COVID‐19. Looking forward, the RSV model we have developed for South Africa could be expanded to explore the impact of new interventions to mitigate RSV burden in this and other LMIC settings.

## AUTHOR CONTRIBUTIONS

**Samantha J. Bents:** Conceptualization; formal analysis; investigation; methodology; project administration; validation; visualization; writing—original draft; writing—review and editing. **Cécile Viboud:** Conceptualization; methodology; project administration; visualization; writing—review and editing. **Bryan T. Grenfell:** Conceptualization; writing—review and editing. **Alexandra B. Hogan:** Formal analysis; methodology; writing—review and editing. **Stefano Tempia:** Conceptualization; writing—review and editing. **Anne von Gottberg:** Data curation; writing—review and editing. **Jocelyn Moyes:** Data curation; writing—review and editing. **Sibongile Walaza:** Data curation; writing—review and editing. **Chelsea Hansen:** Methodology; writing—review and editing. **Cheryl Cohen:** Conceptualization; project administration; writing—review and editing. **Rachel E. Baker:** Conceptualization; formal analysis; methodology; writing—review and editing.

## CONFLICT OF INTEREST STATEMENT

Anne von Gottberg, Cheryl Cohen, Jocelyn Moyes, and Sibongile Walaza acknowledge grants from Sanofi Pasteur, PATH, Bill & Melinda Gates Foundation, and SA‐Medical Research Council, The Centers for Disease Control and Prevention (US), and Wellcome Trust. Alexandra B. Hogan was previously engaged by Pfizer Inc. to advise on modeling RSV vaccination strategies for which she received no financial compensation. Ms. Hansen received contract‐based hourly fees from Sanofi outside of the submitted work.

### PEER REVIEW

The peer review history for this article is available at https://www.webofscience.com/api/gateway/wos/peer-review/10.1111/irv.13229.

## ETHICS STATEMENT

The Human Research Ethics Committee (HREC) of the University of Witwatersrand and the Human Biomedical Research Ethics Committee (BREC) of the University of KwaZulu‐Natal gave ethical approval for the SARI protocol included in this work, protocol numbers M081042 and BF157/08, respectively. The Human Biomedical Research Ethics Committee (BREC) of the University of KwaZulu‐Natal gave ethical approval of the ILI protocol included in this work, protocol number BF080/12.

## Supporting information


**Figure S1.** Supporting Information.
**Figure S2.** Supporting Information.

## Data Availability

Data are available on request from the authors.
