# Supplementary material for: Modeling the impact of COVID‐19 nonpharmaceutical interventions on respiratory syncytial virus transmission in South Africa
Source: Influenza Other Respir Viruses. 2023 Dec 10;17(12):e13229. doi: 10.1111/irv.13229 (PMC10710953; doi:10.1111/irv.13229)
Supplement: Supplementary file 1 — Figure S1. Supporting Information. Figure S2. Supporting Information. [file IRV-17-e13229-s001.docx]

Supplementary Information for: Modeling the impact of COVID-19 nonpharmaceutical interventions on RSV transmission in South Africa

Bents et al.

Supplementary Figure 1
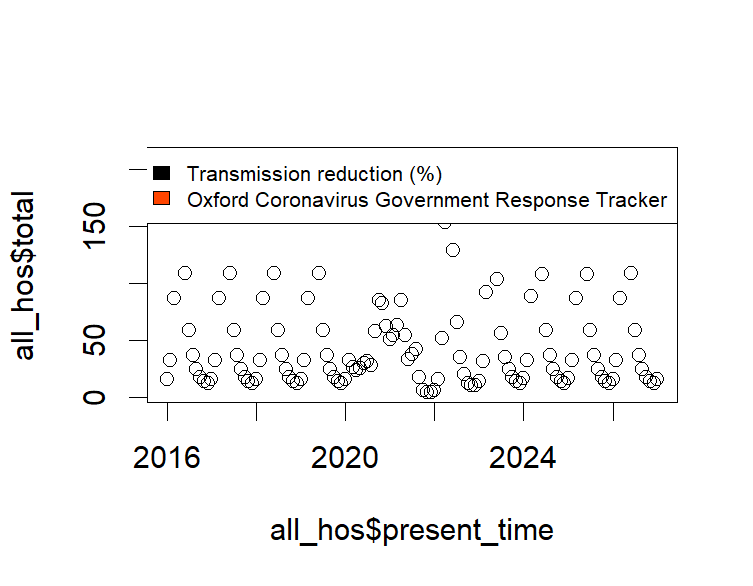


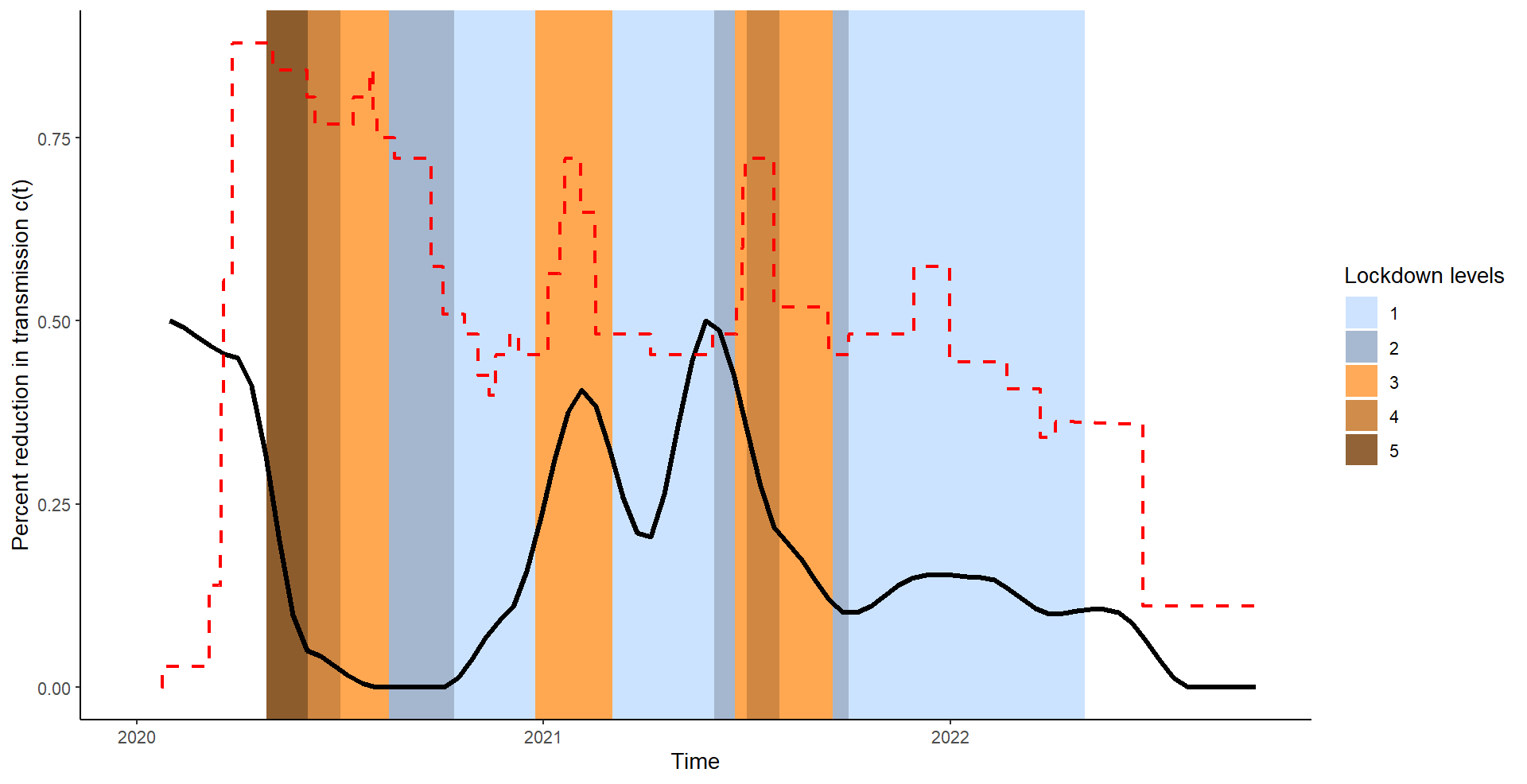


In South Africa, a 5-level alert system was put in place by the government in March of 2020 to determine the use of NPIs in limiting COVID-10 transmission. NPIs were instituted based on perceived COVID-19 national risk, and included social distancing, travel bans, school closures, mask wearing, and other government measures. The timing of the government alerts is shown by the colored bars. We compare the percent reduction in transmission *c(t)* fit from the data (black line) to the Oxford Coronavirus Government Response Tracker (dashed line), which estimates an aggregate stringency score based on various NPI response indicators. A score of 1 indicates highest stringency.

Supplementary Figure 2


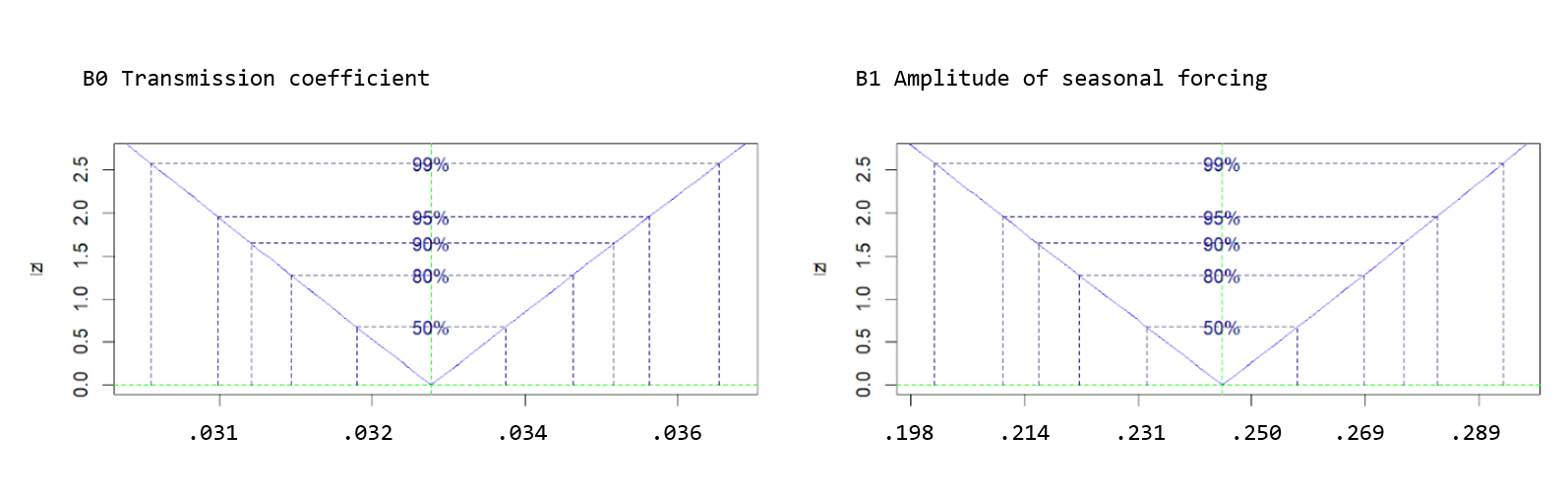


In the first fitting step, we use maximum likelihood to estimate four model parameters. Here we show fitting results with 95% confidence intervals for seasonal forcing and amplitude of transmission parameter estimation.
